# Cardiac damage after polytrauma: the role of systematic transthoracic echocardiography - a pilot study

**DOI:** 10.1186/s13017-025-00596-5

**Published:** 2025-03-11

**Authors:** Larissa Sztulman, Aileen Ritter, Roberta de Rosa, Victoria Pfeiffer, Liudmila Leppik, Lewin-Caspar Busse, Elena Kontaxi, Philipp Störmann, René Verboket, Elisabeth Adam, Ingo Marzi, Birte Weber

**Affiliations:** 1 Department of Trauma Surgery and Orthopedics, Goethe University Frankfurt, University Hospital, 60590 Frankfurt, Germany; 2 Department of Cardiology, Goethe University Frankfurt, University Hospital, 60590 Frankfurt, Germany; 3 Department of Anaesthesiology, Intensive Care Medicine and Pain Therapy, Goethe University Frankfurt, University Hospital, 60590 Frankfurt, Germany

**Keywords:** Multiple trauma, Contusio cordis, Cardiomyopathy, Troponin T, Traumatic valvular dysfunction, Echocardiography

## Abstract

**Background:**

Heart injuries following polytrauma (PT) are identified as a predictor of poor outcome. The diagnostic algorithm of cardiac damage after trauma consists of the systemic measurement of cardiac damage markers, a 3-channel ECG and if there are any suspicious findings, the conduction of a transthoracic echocardiography (TTE). The aim of this study was to implement a systematic analysis of cardiac function using TTE in PT-patients.

**Methods:**

This study is a prospective non-randomized study, conducted in a German Level 1 Trauma Centre between January and July 2024. All polytraumatized patients with an ISS ≥ 16 were included immediately after entering the emergency department. Blood samples were withdrawn at 6 timepoints, at the Emergency room, 24 h, 48 h, three, five and ten days after admission to the hospital. Cardiac damage was measured by Troponin T (TnT) ECLIA, as well as NT-proBNP measurements. Entering the intensive care unit, transthoracic echocardiography was performed at two time points (day 1 and 2), by an experienced Cardiologist.

**Results:**

During the pilot phase, cardiac contusion was detected in 14.3% of patients, with significantly elevated TnT levels on arrival, after 24 (**p ≤ 0.01) and 48 h (*p ≤ 0.05) compared to patients without cardiac contusion. Echocardiographic findings revealed that 25% of all patients had wall motion abnormalities, and 20% showed relaxation disorders. Right ventricular function, measured by TAPSE (tricuspid annular plane systolic excursion), RVEDD (right ventricular end diastolic diameter) and sPAP (systolic pulmonary arterial pressure), was slightly impaired in trauma patients, while the left ventricular function (ejection fraction (EF) and left ventricular end diastolic diameter (LVEDD)) was preserved. We observed the increase of TnT and an increase of the heart failure marker NT-proBNP over the time. These biomarkers were associated with pre-existing cardiac risk factors, the ISS and changes in the right or left ventricular function. Mitral valve insufficiency (grade 1) was present in 50% and tricuspid valve (grade 1) insufficiency in 30%.

**Conclusions:**

Taken together, we conducted for the first time of our knowledge, a systematic TTE analysis in PT-patients. We observed a slightly reduced right ventricular function, as well as mitral and tricuspid valve regurgitations in the patients.

**Supplementary Information:**

The online version contains supplementary material available at 10.1186/s13017-025-00596-5.

## Introduction

Heart injuries following multiple trauma are identified as a predictor of poor outcome with a prolonged ventilation interval as well as a longer in-hospital stay of these patients [1, 2]. In a post-mortem analysis of polytraumatized patients, macroscopic heart injuries were observed in 18% of he patients, who died at the scene and only 1% of atients admitted to the hospital. These findings suggest that macroscopic heart injury is immediately life-threatening at the scene [3]. An involvement of the heart injury can be present with a wide variation of symptoms like dysrhythmias, ventricular fibrillation, impaired cardiac function or sudden cardiac arrest [4, 5]. Most often blunt cardiac injury is associated with thoracic trauma and occurs with an incidence of 10 to > 70% [6, 7]. According to the German Society of Trauma Surgery (DGU), 29.3% of the polytrauma-atients in 2022 (initial AIS ≥ 3) showed a chest trauma (*n* = 76 893 polytraumatized patients) [8]. In patients with severe blunt chest trauma, 37.5% presented an impairedleft ventricular stroke work index (LVSWI) after 12 h. This early depression of cardiac function was associated with a poor outcome of these patients: 78% died in the course [2].

Due to the variety of symptoms, diagnosing cardiac dysfunction or any kind of cardiac damage might be challenging. The diagnostic algorithm of cardiac damage after trauma consists of the systemic measurements of cardiac damage markers like troponin, a 3-channel ECG and if there are any clinically suspicious findings, the conduction of a transthoracic echocardiography [9, 10]. In the present study, we defined cardiac damage as either an increase in troponin concentration or/and impairment of cardiac function, as measured by TTE. After polytrauma, the serum troponin concentrations have been correlated with the Injury Severity Score (ISS) and the mortality due to polytrauma [3]. Although troponin has some limitations as a marker of cardiac damage in severely injured patients—such as its dependence on kidney function—no superior biomarker has been identified so far [11, 12]. Therefore, high sensitivity TnT measurement in the emergency department remains the gold standard for laboratory diagnostics in multiple injured patients. A 3 channel ECG is further part of the diagnostic algorithm of blunt cardiac trauma. Non-specific dysrhythmias like a sinus tachycardia, are common after trauma and have been observed in up to 80% of patients [6]. Chen et al. (2021) reviewed the use of ECG after traumatic brain injury (TBI) and found that, although its utilization rate is very low, it was associated with decreased in-hospital mortality. Therefore, monitoring cardiac electrical activity may improve outcomes for TBI patients [13]. However, conduction of an ECG can also be misleading, as it was shown in a case report of a 37-years-old male patient, after motor vehicle accident: the emergency physician on the scene diagnosed a myocardial infarction by ST-elevation, although the hemodynamic instability of this patients was based on a massive cardiac and pulmonary contusion [14]. In such cases, the TTE or transoesophageal echocardiography should be added to the standard algorithm.

Although recommended in the S3 polytrauma guidelines by the German Society for Emergency Surgery, echocardiographic measurements in polytraumatized patients are rarely performed and generally underutilized. Echocardiography is a useful tool to assess and monitor any cardiovascular or hemodynamic changes, for example the left ventricular function or the volume load of a trauma patient. Furthermore, it is an easily accessible, non-invasive and rapid bedside assessment tool to exclude any fatal cardiac pathologies such as cardiac tamponade, rupture of the coronary arteries and valvular dysfunction [15–17]. In an experimental porcine model of polytrauma, our working group previously described changes in the left ventricular contraction by measuring a reduced ejection fraction (EF) and shortening fraction (SF) in pigs 24 h after chest trauma, liver laceration, femur fracture and haemorrhagic shock [18]. Further the development of traumatic valvular damage was observed 6 h after polytrauma in pigs [19]. Caused by the location of the right atrium and the right ventricle within the mediastinum, the right heart is the most common injured part of the heart [6]. The current literature on contusio cordis primarily consists of rare case reports detailing valvular injuries resulting from trauma. For example, a 38-year-old woman who experienced a severe blunt chest trauma from a car accident required tricuspid valve replacement with a bioprosthesis due to acute severe tricuspid regurgitation and hepatic vein reflux [20]. Late appearance of cardiac damage could be adequately diagnosed by echocardiography. For instance, a 58-year-old man developed symptoms of heart failure 10 days after a car accident. This was associated with rupture of tendinous chords leading to significant tricuspid regurgitation, as well as pericardial rupture with cardiac herniation [21]. Overall, the potential of echocardiography in the detection of cardiac damage after trauma is enormous, yet systematic analysis of cardiac function is seldomly conducted.

Therefore, the aim of the present study was to establish a standardized approach for analysing cardiac function via transthoracic echocardiography in polytraumatized patients, with the goal of detecting myocardial damage and assessing cardiac dysfunction in critically injured patients.

## Materials and methods

### Ethical approval

Ethical approval was granted by the local ethics committee of the University of Frankfurt (approval ID 89/19). Written informed consent was acquired from all enrolled patients.

### Study design

The present study is a prospective observational study, conducted in a German Level 1 Trauma Centre. All polytraumatized patients with an ISS ≥ 16 who were admitted to the emergency department between January and July 2024 were included in the study upon arrival (*n* = 35). Blood withdrawal was carried out during routine diagnostic at 6 timepoints (admission to the Emergency room, 24 h, 48 h, 3 days, 5 days and 10 days after admission to the hospital). Blood samples were analysed during routine laboratory measurements. Additionally, samples were kept on ice and plasma was separated by centrifugation at 3500×g, 15 min 4 °C, according to the NTF Biobank protocol [22]. Clinical and demographic data were retrieved from the digital patient records. Results were then compared to normal reference values from the literature and routine laboratory information.

### TTE

Upon admission to the intensive care unit, transthoracic echocardiography was performed at two time points by an experienced Cardiologist. Such examination parameters as ejection fraction (EF), left ventricular end-diastolic diameter (LVEDD), thickness of the interventricular septum and posterior wall of the left ventricle (IVS/LVPW), tricuspid annular plane systolic excursion (TAPSE), right ventricular end-diastolic diameter (RVEDD) and systolic pulmonary artery pressure (sPAP) were assessed. Additionally, observations were made for pericardial or pleural effusion, valvular regurgitation, dyskinesia, and relaxation disorders. The measurements were conducted in cooperation with the Department of Cardiology of the University in Frankfurt in accordance with the general echocardiography standards of the German Centre for Cardiovascular Research (DZHK). For the echocardiographic examination, the same devices were consistently used (Manufacturer: GE HealthCare, models: Venue Go R2 and Vivid iq).

### Cardiac damage markers

Serum TnT and NT-pro BNP concentrations were measured via highly sensitive electrochemiluminescence immunoassays (ECLIA, Roche, Rotkreuz, Switzerland).

### Statistical analysis

All statistical analysis were conducted with Graph Pad-Prism 9 (Dotmatic, San Diego, CA, USA). Data were analysed via a Kruskal–Wallis test followed by Dunn‘s multiple comparisons test. For the statistical analysis of two groups, the Mann–Whitney test was performed. Correlation analysis was carried out with the spearman rank correlation. A moderate correlation was defined 0.7 ≥ *r* ≥ 0.5; a strong correlation was determined if *r* ≥ 0.7. Analysis with a p-value ≤ 0.05 were considered significant. Data are presented as mean ± standard error of the mean (SEM).

## Results

### Patient characterisation

The present pilot study included 35 polytraumatized patients with a mean ISS of 29.1 ± 9.5, directly admitted to the German Level 1 Trauma centre in 2024. In these patients, cardiac damage markers and heart failure markers were measured over 10 days. Systematic transthoracic echocardiography was conducted within 24 h and 48 h after admission. As demonstrated in Tables 1 and 77% of the included polytrauma patients were male. The average age was 55.8 years and approximately one fourth were non-survivors (25.7%). In the context of cardiac damage in trauma patients, it is important to note that 65.7% were diagnosed with a chest trauma (2.9% open chest trauma), while 14.3% of all patients suffered a contusio cordis defined by troponin increase or CT diagnostics. Moreover, one fourth of the patients required resuscitation (25.7%) and over 30% developed arrythmias during the ICU stay (31.42%). Almost 20% of included patients jumped/fell from a great high, 14.3% were motorcycle accidents or tripping falls; car accidents were the trauma mechanism in 11.4%, 8.6% were falls down the stairs, accidents as pedestrian, work accidents and patients captured by a subway, while 5,7%had other trauma mechanism like knife attacks.

**Table 1:** Demographic data of study patients

Parameter	Mean value ± SD
ISS	29.1 ± 9.5
Sex [male: female in %]	77.1: 22.9
Age [years]	55.8 ± 20.1
Height [cm]	172.4 ± 10.3
Weight [kg]	81.6 ± 17.3
BMI	29.7 ± 8.3
Contusio cordis [%]	14.3
Score2 [%]	8.7 ± 10.3
Non-Survivors [%]	25.7
Previous LAE [%]	0
Previous Arrythmia [%]	8.6
Previous Coronary Artery Disease [%]	8.6
Arrythmia in ER [%]	22.9
Reanimation in ER [%]	25.7
Chest trauma (blunt– open) [%]	65.7 (open 2.9%)
Sternal fracture [%]	14.3
Smoker [%]	17.1
Arterial hypertension [%]	42.9
Diabetes mellitus [%]	8.6
Hyperlipidaemia [%]	14.3
Past myocardial infarction [%]	11.42
NYHA (heart failure) [%]	2.9
Angina pectoris in the last 4 weeks [%]	5.7
Arrhythmia during ICU stay [%]	31.42
LAE during ICU stay [%]	8.6
Reanimation during ICU stay [%]	20
Infection during ICU stay [%]	45.7%
Shock (cardiac/hemorrhagic/septic/unknown)	34.3% (8.6%/20.0%/2.9%/2.9%)

Data are presented as Mean ± standard deviation (SD), (*n* = 35)

### Cardiac damage evaluations via laboratory markers

**Fig. 1:**
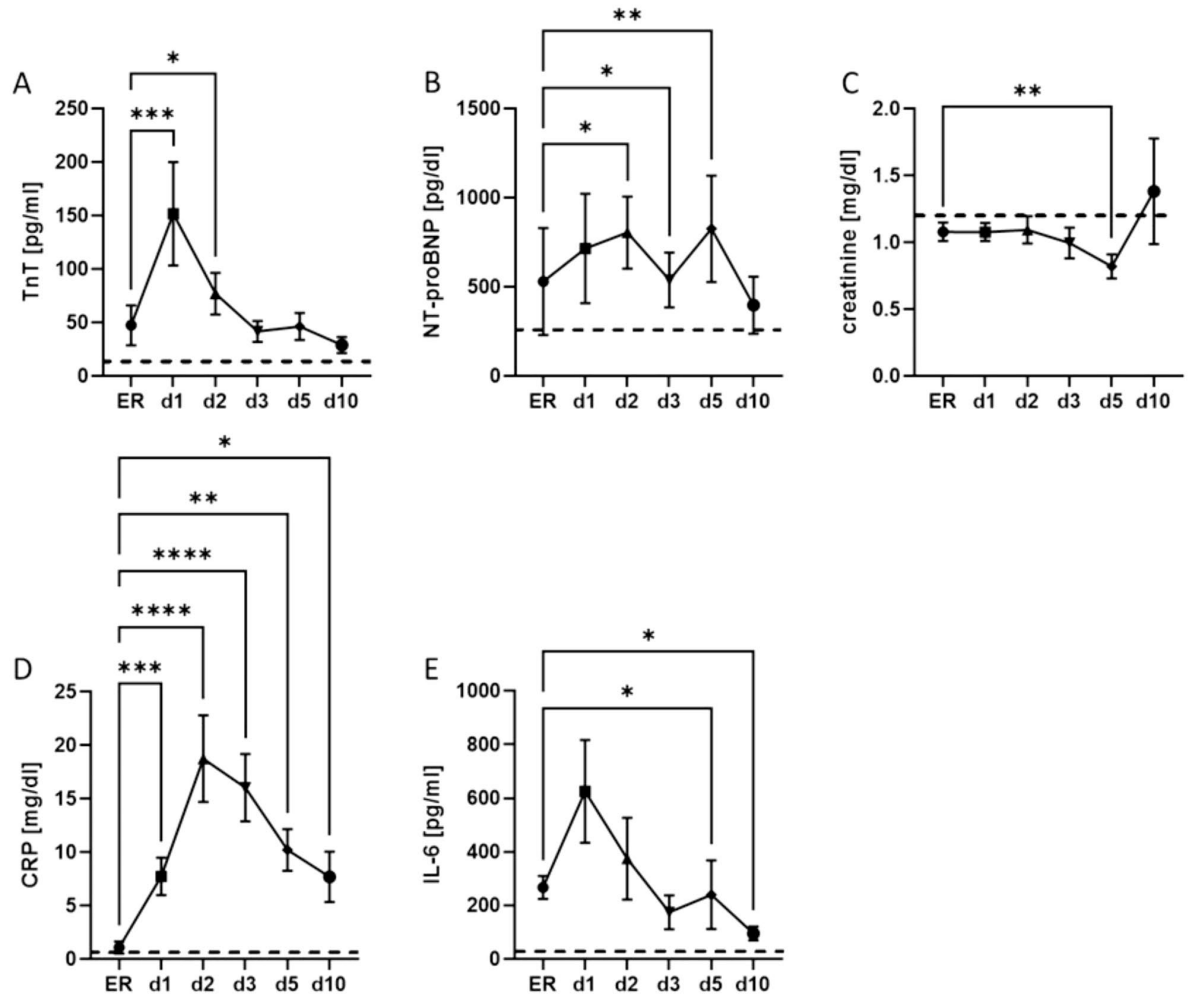
Systemic biomarkers expression in plasma of polytrauma patients 10 days over trauma. **A**) Systemic concentration of troponin T (TnT) in polytrauma patients in the emergency room (ER) and on days 1, 2, 3, 5 and 10 after trauma in comparison to standard values (**C**I 10.73 to 250.4). **B**) Levels of N-terminal prohormone of brain natriuretic peptide (NT-proBNP) in pg/dl over 10 days after polytrauma compared to standard values (CI -93.10 to 1348). **C**) Creatinine levels in mg/dl as marker of kidney function in the polytrauma collective (CI 0.556 to1.301). Inflammatory routine biomarkers C-reactive protein (CRP) (CI -0.07222 28.08) to and Interleukin-6 (IL-6) during the first 10 days after severe trauma with an Injury Severity Score ≥ 16 (CI -22.72 to 1016). Dashed line = standard values, d1 = day 1, d2 = day 2, d3 = day 3, d5 = day 5 and d10 = day 10. *n* = 35, *p ≤ 0.05, **p ≤ 0.01, ***≤0.001, ****p ≤ 0.0001

First, we quantified cardiac damage after polytrauma via laboratory markers. A significant increase (*p* ≤ 0.05) in the cardiac damage marker TnT was detected in polytrauma patients one day after trauma, with levels already rising in the ER compared to standard values (Fig. 1A). Furthermore, we analysed a classical heart failure marker NT-proBNP in the trauma patients. It was significantly (*p* ≤ 0.05) elevated in polytrauma patients on day 2, 3 and 5 compared to measurements taken in the ER (Fig. 1B). A well-known limitation of Troponin T is its dependence on renal function. Therefore, we measured creatinine levels, which did not show any impairment in polytrauma patients compared to standard values during the first 10 days after trauma (Fig. 1C).

To assess post-traumatic inflammation in polytrauma patients, C-reactive protein (CRP) and Interleukin-6 (IL-6) were measured in the routine laboratory assessment. CRP levels were not elevated in the ER but begun to rise one day after trauma, reaching peak concentration on day two and being significantly elevated on all days (*p* ≤ 0.05, D, Fig. 1). The levels of IL-6, a biomarker that reacts more quickly to inflammation, are shown in Fig. 1E. IL-6 was already significantly increased in the ER (*p* ≤ 0.0001), reached its peak on day 1 and stayed significantly elevated until day 5 (*p* ≤ 0.0001, Fig. 1E).

### Cardiac damage evaluations via echocardiography

**Fig. 2:**
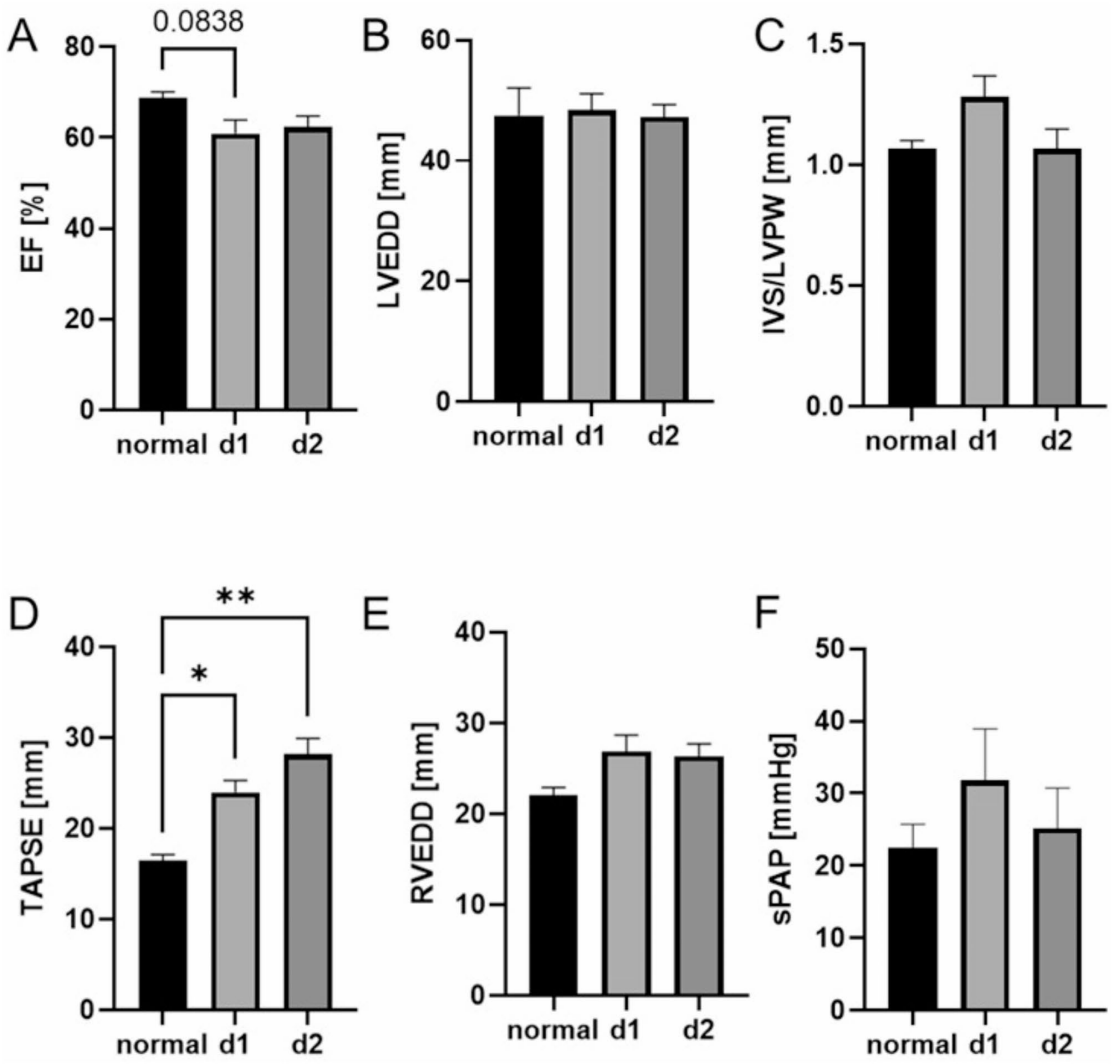
Transthoracic echocardiography measurements of the right and left ventricle function after polytrauma. **A**) Measurements of ejection faction (EF) in % at day 1 and day 2 after polytrauma in comparison to standard values (CI 54.22 to 72.73). **B**) left ventricular end-diastolic diameter (LVEDD) as additional parameter of the left ventricle function in comparison to standard measurement values (CI 32.95 to 62.05). **C**) Thickness of the interventricular septum and posterior wall of the left ventricle (IVS/LVPW) at both timepoints as parameter of left ventricle hypertrophy (CI 0.89 to 1.477). To evaluate the right ventricle function: **D**) The tricuspid annular plane systolic excursion (TAPSE) (CI 14.45 to 31.94), as well as **E**) the right ventricular end-diastolic diameter (RVEDD) (CI 19.09 to 31.17) and **F**) the systolic pulmonary artery pressure (sPAP) were compared with standard values (CI 10.83 to 51.65). Normal = standard values d1 = day 1, d2 = day 2, *p ≤ 0.05, **p ≤ 0.01

Next, we verified cardiac damage in polytrauma patients via transthoracic echocardiography performed on days one and two after trauma. As parameters of the left ventricle function, we measured the EF and LVEDD. By trend, EF was reduced in trauma patients (*p* = 0.08) at days one and two after trauma (Fig. 2A). The LVEDD did not showed any significant changes at both measurement time points (Fig. 2B). To further assess potential left ventricle hypertrophy, we measured the ratio of interventricular septum (IVS) and left ventricular posterior wall (LVPW). In the present patient collective, a mild hypertrophy of the left ventricle was observed at day one after multiple trauma (Fig. 2C). For evaluation of the right ventricle, we measured the TAPSE, the RVEDD and the sPAP. RVEDD was not significantly altered at either time point (Fig. 2E), while TAPSE was significantly increased one day after trauma (*p* ≤ 0.05) and showed a further increase on day 2 (*p* ≤ 0.01) (Fig. 2D). SPAP, a potential marker of pulmonary hypertension, was slightly elevated in polytrauma patients; however, this elevation was not statistically significant (Fig. 2F).

**Fig. 3:**
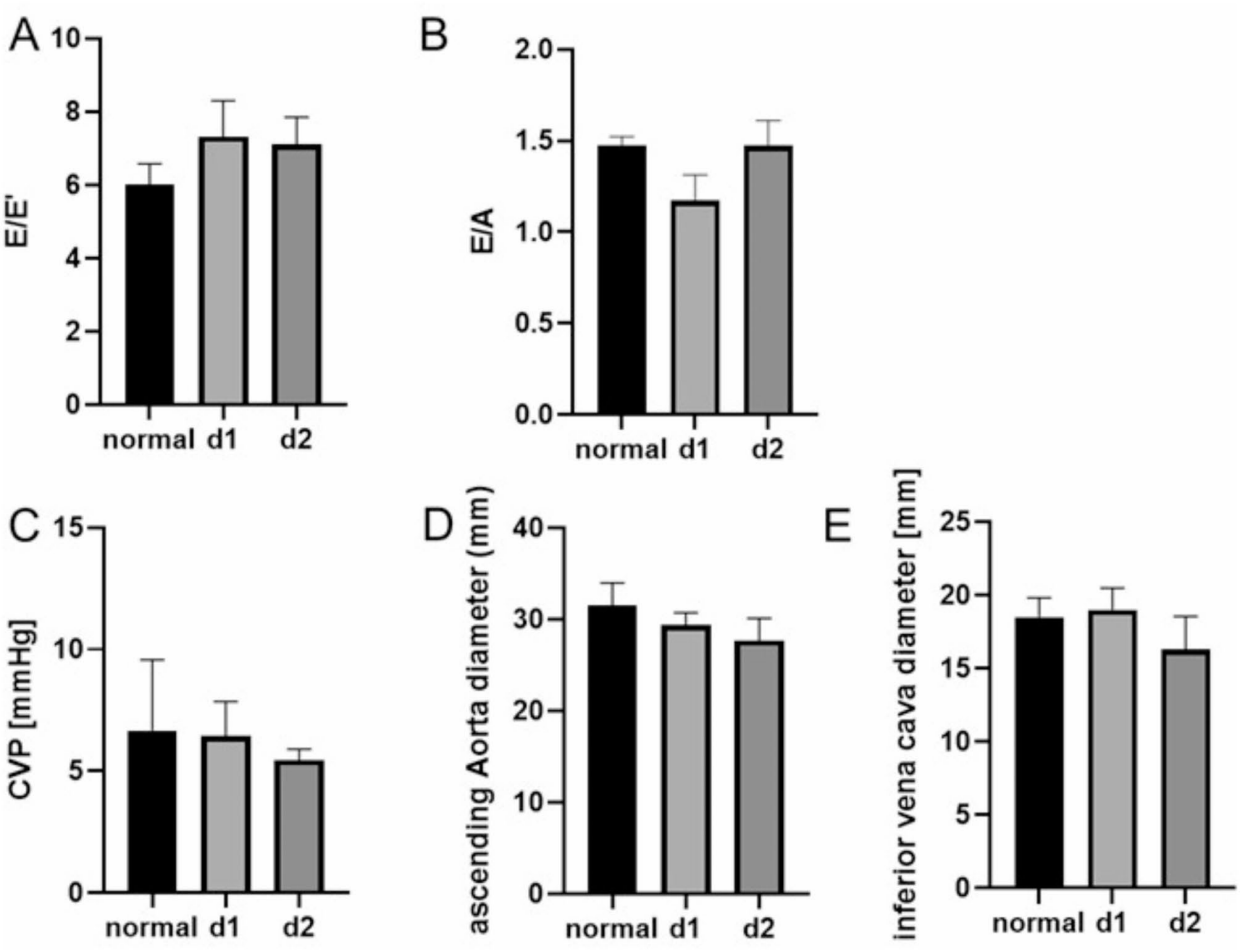
Diastolic dysfunction after polytrauma. As parameter of the diastolic dysfunction, the E/E’ ratio (**A**) (CI 3.516 to 9.643) and the E/A ratio (**B**) was measured at two timepoints after severe trauma and compared with standard values (CI 1.169 to 1.776). The central venous pressure (CVP) (**C**) in mmHg (CI -5.837 to 19.17), the diameter of the ascending aorta (**D**) (CI 22.29 to 39.56) and the diameter of the Vena cava inferior (**E**) in mm were additional parameters assessed during transthoracic echocardiography (CI 11.10 to 23.12). Normal = standard values d1 = day 1, d2 = day 2

As additional parameters of potential diastolic dysfunction, E/E’ and the E/A ratios were monitored at both measurement timepoints. E/E’ was slightly increased at days one and two after trauma (Fig. 3A), while E/A was by-trend reduced at day one after trauma (Fig. 3B). Central venous pressure (CVP) (Fig. 3C), the diameter of the ascending aorta (Fig. 3D) and the diameter of the inferior vena cava (Fig. 3D) showed no significant difference in comparison to standard parameters.

To provide an overview of the extensive information obtained from transthoracic echocardiography, we have summarized all relevant results in Table 2. 20 of the included patients received echocardiography on days one and two. 45% of these patients developed pleural effusion, while 15% developed pericardial effusion. As a therapeutic measure, 20% of all patients needed a chest tube to relief hematoma, or a pneumothorax. Regarding cardiac damage, it is notable that 20% of polytrauma patients experienced disturbed myocardial relaxation, and 50% of those with this condition died.

Another noteworthy observation is that half of the polytrauma patients exhibited minimal (grade one) mitral valve regurgitation within 24 h, with an additional 5% showing this condition after 48 h. The aortic valve does not appear to be as vulnerable as the mitral or the tricuspid valve, with only 5% of the patients developing minimal (grade one) aortic valve regurgitation. The minimal (grade one) tricuspid valve regurgitation was observed in 30% of patients after 24 h and in 45% after 48 h (Table 2).

**Table 2:** Transthoracic echocardiographic findings in polytrauma patients, one and two days after trauma. EF = Ejection fraction, lvedd = left ventricular end-diastolic diameter, ivs/lvpw = thickness of the interventricular septum and posterior wall of the left ventricle, tapse = tricuspid annular plane systolic excursion, rvedd = the right ventricular end-diastolic diameter and sPAP = systolic pulmonary artery pressure (*n* = 20)

Parameter	Day one	Day two	Normal values
Pleural effusion (% of patients)	45%	45%	
Pericardial effusion (% of patients)	15%	15%	
Disturbed myocardial relaxation (non-survivors of these patients), (% of patients)	20% (50%)	20% (50%)	
Chest Tube, (% of patients)	20%	20%	
Arrhythmia, (% of patients)	15%	15%	
Wall movement disorder, (% of patients)	25%	25%	
Aortic valve regurgitation, (% of patients)			
- minimal/Grade 1 - Grade 2	5% 0%	5% 0%	
Mitral valve regurgitation, (% of patients) - Minimal/Grade 1 - Grade 2	50% 0%	55% 10%	
Tricuspid valve regurgitation, (% of patients) - Minimal/Grade 1 - Grade 2	30% 10%	45% 15%	
EF [%]	61 ± 9.1	62 ± 9.6	> 60
LVEDD [mm]	48 ± 8.2	48 ± 6.9	36–55
IVS/LVPW [mm]	1.3 ± 0.3	1.1 ± 0.2	IVS ≤ 12 mm/ LVPW ≤ 12 mm
TAPSE [mm]	24 ± 3.7	28 ± 6.8	> 17
RVEDD [mm]	25 ± 7.2	26 ± 5	< 30
E/E’	7.3 ± 2.6	6.8 ± 2.7	< 8
E/A	1.2 ± 0.4	1.4 ± 0.5	1–2
sPAP [mmHg]	32 ± 14	25 ± 12	< 36

### Correlation between biomarkers, cardiac risk factors and transthoracic echocardiography measurements

**Fig. 4:**
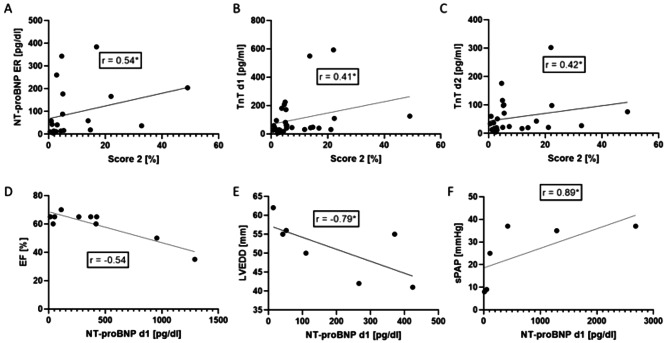
Correlation analysis with cardiac risk and NT-pro-BNP. **(A**) moderate correlation between N-terminal prohormone of brain natriuretic peptide (NT-proBNP) at the ER with cardiac risk Score 2 (*r* = 0.54; CI 0.1302 to 0.7940). Moderate Correlation between 10-year cardiac risk score and the troponin T concentration on day 1 (*r* = 0.41; CI 0.04792 to 0.6855) (**B**) and day2 (*r* = 0.42; CI 0.004517 to 0.6890) (**C**). (**D**) The ejection fraction (**EF**) in % correlated moderately with the levels of NT-proBNP (*r*=-0.54; CI-0.9760 to -0.6189). A stronger correlation between NT-proBNP and the left ventricular end-diastolic diameter (LVEDD) (*r*=-0.79; CI -0.9498 to 0.1292) (**E**) and further with the sPAP was strongly positively correlated with levels of NT-proBNP (*r* = 0.89; CI-0.3093 to 0.9598) (**F**)

To further analyse the significance of our results, we conducted a correlation analysis between biomarkers, cardiac risk factors and transthoracic echocardiography measurements. Cardiac risk factors were calculated via Score 2 [23] based on such parameters as blood pressure, HDL and cholesterol concentrations, sex, age, smoking. We correlated calculated Score 2 risk values with the measured levels of NT-proBNP and troponin T in the polytrauma patients’ collective. As presented in Fig. 4, NT-pro-BNP mildly correlated with the Score 2 (Fig. 4A, *r* = 0.54), while troponin levels measured on day 1 and 2 were associated with the Score 2 (Fig. 4B and C). Further, we performed correlation analysis of different parameters measured by mean of echocardiography and NT-proBNP. First of all, a mild negative correlation of the EF and NT-proBNP on day one was shown (Fig. 4D, *r*=-0.54). At the same time point the strong negative correlation was observed between LVEDD and NT-proBNP (Fig. 4E, *r*=-0.79). Interestingly, the strongest correlation was observed between NT-proBNP and the sPAP (Fig. 4F, *r* = 0.89).

**Fig. 5:**
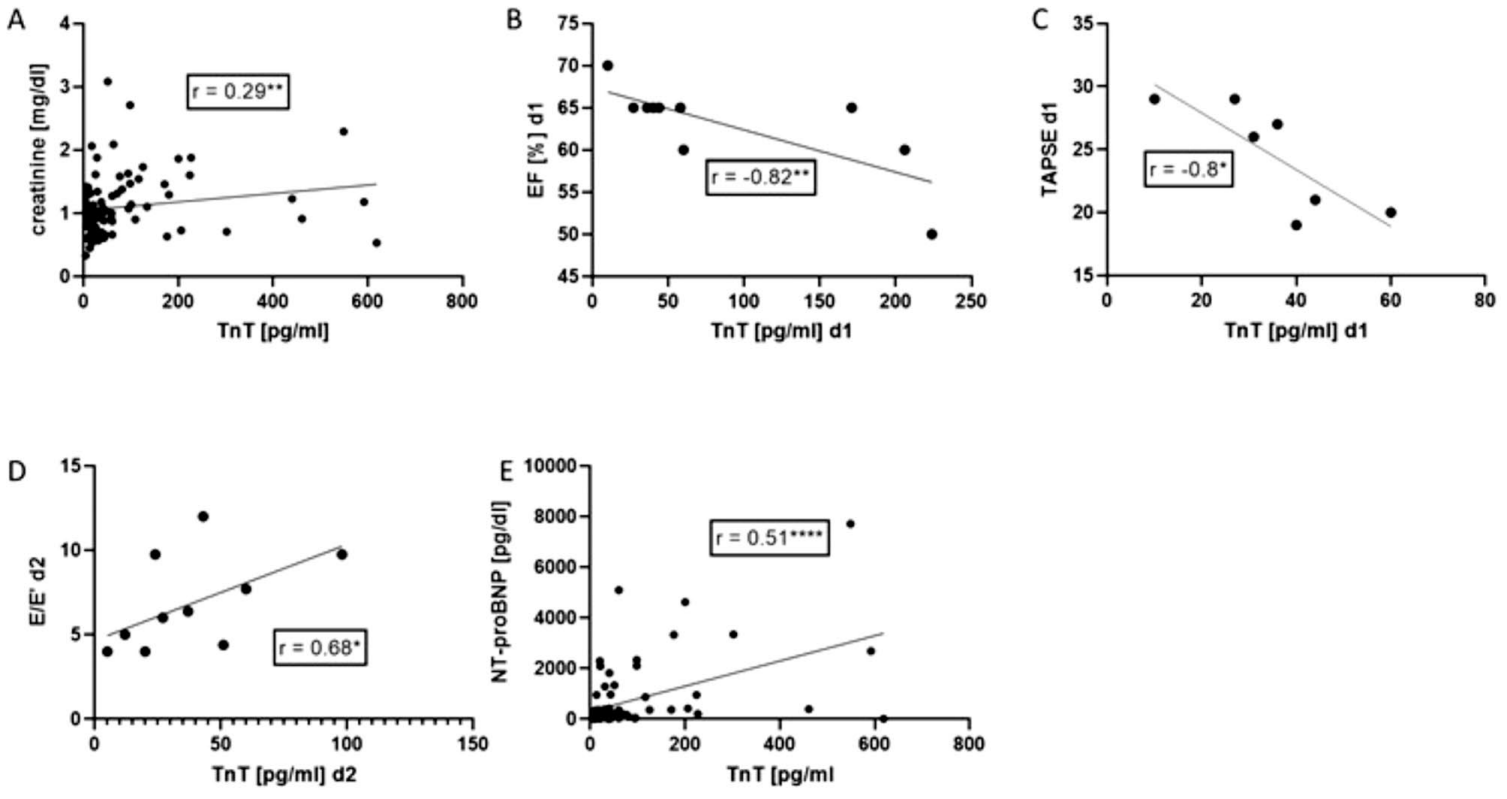
Correlation analysis with acute myocardial damage marker troponin T (TnT). **(A**) Correlation analysis of TnT in pg/ml with creatinine in mg/dl (*r* = 0.29; CI 0.05561 to 0.4452). (**B**) A strong negative correlation between TnT and the ejection fraction (EF) was detected one day after trauma (*r*=-0.82; CI -0.8489 to 0.1729). (**C**) Negative correlation with right ventricle function marker tricuspid annular plane systolic excursion (TAPSE) (*r*=-0.8; CI -0.8322 to 0.9181). (**D**) A moderate positive correlation exists for troponin and E/E`(*r* = 0.68; CI 0.2465 to 0.9212). (**E**) Correlation analysis of troponin and N-terminal prohormone of brain natriuretic peptide (NT-proBNP) showed a moderate association (*r* = 0.51; CI 0.3061 to 0.6614). *p ≤ 0.05, **p ≤ 0.01, ****p ≤ 0.05. Spearman rank correlation

Next, we performed correlation analysis of TnT with laboratory and echocardiographic parameters. As expected, TnT levels were associated with the renal function measured by creatinine (r = 0.29) (Fig. 5A). The level of troponin at day one negatively correlated with the left ventricle parameter EF (r = -0.82) (Fig. 5B) and the right ventricle parameter TAPSE (r =-0.8) (Fig. 5C). In addition, we observed that at day two troponin T level positively correlated with the diastolic dysfunction parameter E/E’ (*r* = 0.68) (Fig. 5D). At least, a moderate correlation between troponin T and heart failure marker NT-proBNP was found (*r* = 0.51) (E).

### Subgroup analysis

**Fig. 6:**
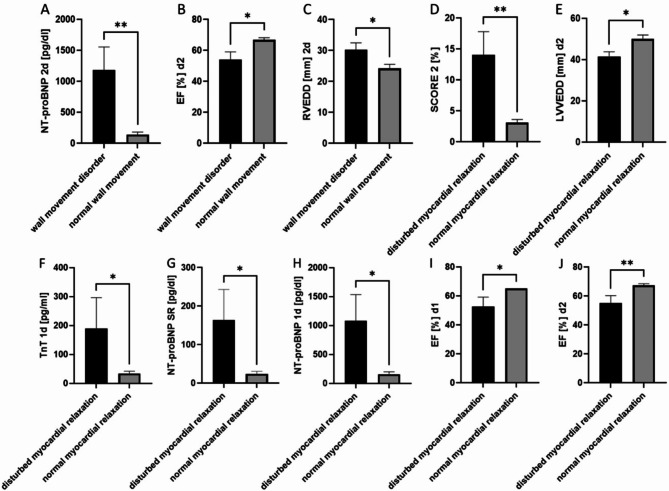
Subgroup analysis of patients with wall movement disorder or disturbed myocardial relaxation. N-terminal prohormone of brain natriuretic peptide (NT-proBNP) at day 2 (**A**) (CI of difference − 2063 to -138.0), ejection fraction (EF) (CI of difference 0.00 to 25.0) (**B)** and right ventricular end-diastolic diameter (RVEDD) at day 2 (**C**) (CI of difference − 12.00 to 0–00) in polytraumatized patients with wall movement disorder compared to patients without normal wall movement. 10 years cardiac risk score 2 (**D**) (CI of difference − 18.90 to -2.3), left ventricular end-diastolic diameter (LVEDD) (**D**) (CI of difference 0.00 to 15.00), troponin T concentration (TnT) at day 1 (**F**) (CI of difference − 543 to 0.00), NT-proBNP concentration at emergency room (ER) (**G**) (CI of difference − 370 to -5.0) or day1 (**H**) (CI of difference − 2267 to -25.0), as well as ejection fraction (EF) at day 1 (**I**) (CI of difference 0.0 to 30.0) and day 2 (**J**) in patients with disturbed vs. normal myocardial relaxation (CI of difference 5.0 to 30.0). *p ≤ 0.05, **p ≤ 0.01. Mann–Whitney test

Further, we divided our patients collective in subgroups based on the presence of wall movement disorder, disturbed function of myocardial relaxation, contusio cordis and chest trauma and survivals and non-survivals. We observed a significantly higher level of day two NT-proBNP in the subgroup of patients with wall movement disorder as compared to the subgroup without (Fig. 6A **p ≤ 0.01). The ejection fraction was significantly higher in patients with normal wall movement (Fig. 6B), while the RVEDD was significantly increased when a wall movement disorder was diagnosed (Fig. 6C).

In the subgroup of patients with a disturbed myocardial relaxation function higher Score 2 was calculated (Fig. 6D), while a normal myocardial relaxation subgroup was characterised by a higher LVEDD (Fig. 6E). Further, elevated TnT levels on day one (Fig. 6F), higher levels of NT-proBNP in the ER (Fig. 6G) and at day one (Fig. 6H) were associated with disturbed myocardial relaxation. The EF was significantly higher in patients with normal myocardial relaxation on days one (Fig. 6I) and two (Fig. 6J).

**Fig. 7:**
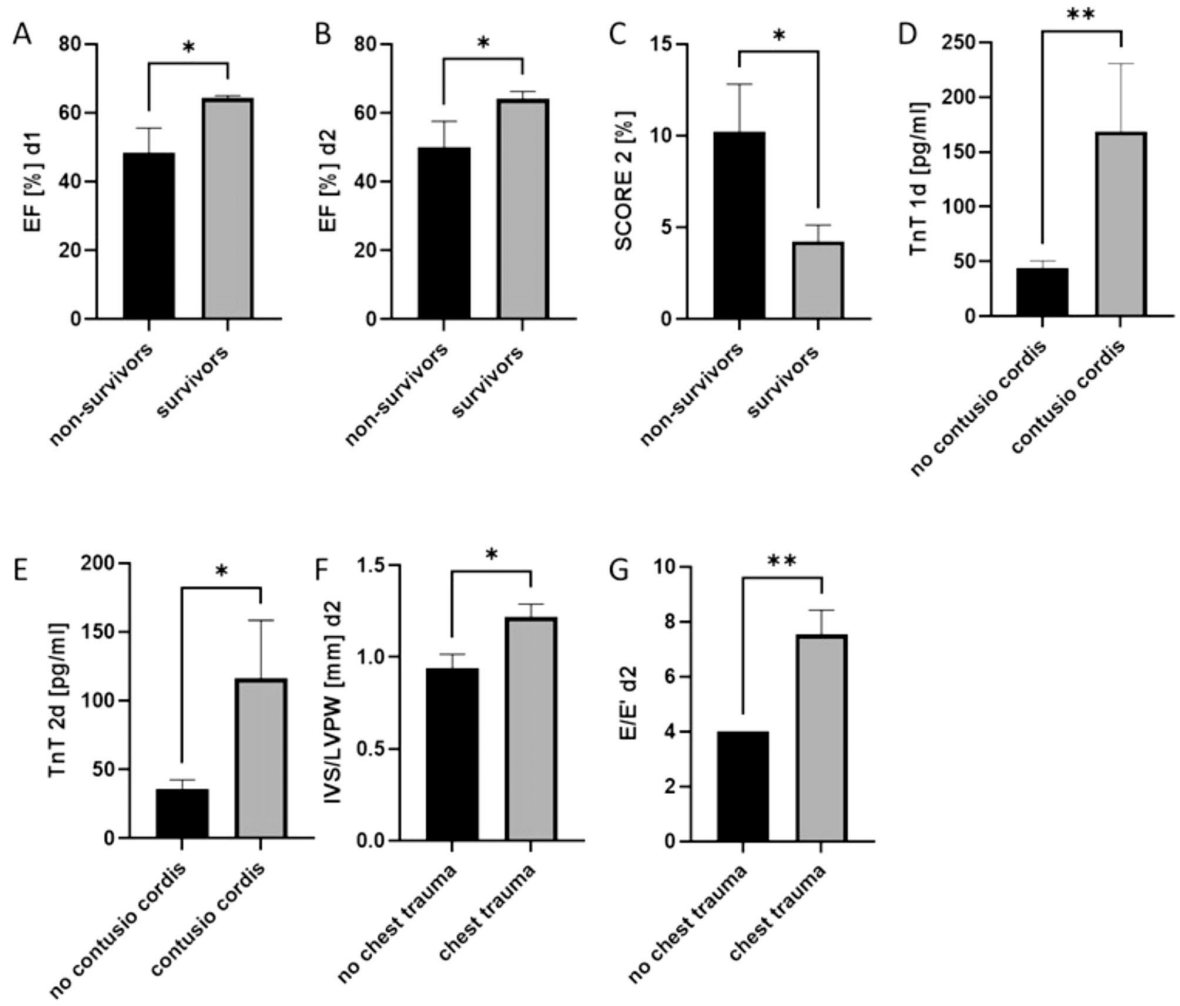
Subgroup analysis of non-survival, contusion cordis or chest trauma. Ejection Fraction (EF) in % at day 1 (**A**) CI of difference 5.0 to 30.0) and day 2 (**B**) in dependency on survival after polytrauma with an Injury Severity Score (ISS) ≥ 16 (CI of difference 5.0 to 30.0). (**C**) Cardiac Risk Score 2 a predictor of a 10-year risk of cardiovascular disease in non-survivors is increased compared to survivors after trauma (CI of difference − 11.6 to -0.30). Troponin T concentration (TnT) in pg/ml at day 1 (**D**) (CI of difference 15.0 to 193.0) and day 2 (**E**) (CI of difference 2.0 to 176.0) after trauma in patients with or without contusion cordis. (**F**) Ratio of interventricular septum (IVS) and left ventricular posterior wall (LVPW) at the end of the diastole determining left ventricular hypertrophy in patients with or without chest trauma at day 2 (CI of difference 0.0 to 0.51). (**G**) Ratio of E/E’ as a marker of left ventricular diastolic dysfunction in the subgroup of polytrauma patients with chest trauma (CI of difference 0.49 to 7.0). *p ≤ 0.05; ** *p* ≤ 0.01. Mann–Whitney test

In comparison among survivors and non-survivors, the EF was significantly decreased at both days in non-survivors, whereas the cardiac risk Score 2 was increased in the patients, who died during the hospital stay (Fig. 7A-C).

In patients with the diagnosis contusio cordis, the TnT concentrations were significantly increased at both measurement time points (Fig. 7D and E). In the patients with chest trauma, the IVS/LVPW ratio was increased (Fig. 7F), and the E/E’ ratio (Fig. 7G) was higher as compared to patients without chest trauma.

## Discussion

### Establishment of the transthoracic echocardiography in polytraumatized patients

The aim of the present pilot study was to establish a systematic analysis of cardiac function via TTE in polytraumatized patients, to detect direct (chest trauma) and indirect (inflammatory) myocardial damage and the impairment of cardiac function in critical traumatized patients. To our knowledge, this is the first systematic analysis of TTE conducted in polytraumatized patients within an ICU setting. In general, implementing a standardized TTE protocol for trauma patients presents several challenges. The initial phase in the emergency department is often highly stressful, leaving limited space and time for a detailed analysis. Therefore, we decided to conduct the first echocardiography measurement within the first 24 h after entering the ICU. Based on previous animal studies [18] and the observation that the EF was reduced within the first 24 h after trauma but normalized thereafter; we decided to conduct a follow-up measurement within 48 h post-trauma. These complex and time-consuming analyses were only feasible in a collaboration of an interdisciplinary team of trauma surgeons, anaesthesiologists and cardiologists. In general, transthoracic echocardiography is a measurement, which should be conducted by a person with years of experience to generate valid measurements. First of all, we tried to train our trauma surgeons in TTE by a course program, but we recognized that a standardized measurement needs a lot more experience, which could not be replaced by a course program. Therefore, support from the department of cardiology was given. We further discussed the protocol of the measurements and decided to standardize the post-trauma protocol in accordance with the general echocardiography standards of the DZHK. These standards include recommendations for the examinations to be included in a structured TTE assessment, and we integrated all these recommended measurements in our protocol. The standardized echocardiography parameters for the systematic measurements are listed in the supplemental Text 1. Another challenge in establishing an echocardiography protocol for trauma patients is the fact that these individuals often present difficult ultrasonic conditions due to factors such as pulmonary issues, positioning on a rotokinetic treatment table, limitations from ventilation, chest tubes, injury patterns, external fixators, or body mass. Therefore, the ultrasound quality was separately documented in all included patients and need to be considered during interpretation of the data.

All in all, such a detailed, systematic analysis of heart function needs a multidisciplinary team and a standard protocol which is examined by an experienced investigator in TTE.

### Detection of cardiac damage in polytrauma via laboratory markers– does troponin T reflect the cardiac damage after trauma?

Next to the establishment of the transthoracic echocardiography, this study measured for the first-time the cardiac damage marker TnT and heart failure marker NT-pro-BNP over 10 days after trauma. TnT levels were significantly elevated on the first day after polytrauma, with a mean value reaching 150 pg/ml. In 2000, the European Society of Cardiology and the American College of Cardiology defined troponin levels above the 99th percentile of the healthy population as a biomarker of significant cardiac damage [24]. As mentioned above, there is a correlation between elevated serum TnT levels in patients after multiple trauma and the ISS, abbreviated injury scale (AIS) of the chest, the survival and the need for catecholamines [3]. Polytrauma patients with elevated levels of TnT in the emergency department, were found to be more likely to require chest tube insertion, exploratory laparotomy, mechanical ventilation, and tracheostomy. Although 18% of polytrauma patients with increased TnT at admission showed no cardiac involvement, these patients had the worst outcome of almost thousand trauma patients in a retrospective study [25]. In the ICU setting, troponin was described as marker of mortality in major trauma [26] and its prognostic value was independent of age, hemodynamic variables and the Glasgow Coma Scale (GCS) [27]. Based on these studies, TnT seemed to be a relevant marker of the outcome in polytrauma patients, which might be mainly influenced by the fact that a severe trauma (high ISS) also led to a distinct release of cardiac damage markers. In these patients the release of cardiac damage markers is partially attributed to direct mechanical impact on the chest, as well as indirect damage resulting from inflammation, which led to cardiomyocyte destruction [9]. However, no study evaluated systematically the relation between TnT levels and the real cardiac function measured by echocardiography in trauma patients yet. This is of broad interest, because troponin release does not necessarily indicate that cardiomyocytes are destroyed. Cardiomyocytes could also release troponin under stress conditions without cell loss and therefore, the heart function might not be completely reflected by the TnT release [28]. One patient in the present study showed extremely high troponin values (up to 1409 pg/ml) with a quite normal left ventricular function (EF of 60%). In the present analysis, TnT was significantly elevated in polytraumatized patients diagnosed with contusio cordis or disturbed myocardial relaxation. The troponin concentration was further correlated negatively with the left ventricular EF and the TAPSE in echocardiographic measurements, as well as positively correlated with the E/E’ ratio, the ISS (Suppl. Figure 1), the heart risk Score 2 and the NT-proBNP concentration. These observations are in accordance with the literature, showing negative correlation between serum troponin I and left ventricular EF in patients with myocardial infarction and acute coronary syndrome [29]. Also, the recovery of the right ventricular function, measured by TAPSE in STEMI patients, was associated with a normalization of TnT concentration, as well as NT-proBNP levels [30]. All in all, the TnT levels in polytraumatized patients seemed to partially reflect the heart function after trauma, measured by EF or TAPSE. Therefore, troponin measurements are playing an important role as screening tool in the diagnostic algorithm for traumatic cardiac damage. However, conditions such as valvular insufficiency or volume overload might not be adequately represented by TnT concentrations, thereby echocardiographic examination seems to be an important additional diagnostic tool in polytrauma patients. Furthermore, we observed individual patients with normal initial troponin concentrations and a strongly impaired cardiac function (e.g. EF of 35%).

Another clinically well-established marker of heart failure is NT-proBNP. Unlike TnT, NT-proBNP is not included in the diagnostic algorithm for polytrauma and is not typically used as a routine marker in the post-traumatic ICU setting. Nonetheless, NT-proBNP is described in the literature as a predictor of the development of atrial fibrillation in general surgical intensive care unit patients, with a cut-off level of 600 ng/l [31]. In multiple trauma patients with an ISS > 16 the serum N-terminal peptide of pro-atrial natriuretic peptide (NT-proANP) and NT-proBNP concentrations were found to be significantly correlated with the clinical signs of multiple organ dysfunction. Further correlations between the levels of serum NT-proANP and NT-proBNP and decreased cardiac index measured by Pulse index Continuous Cardiac Output system were detected [32]. Furthermore, in patients with major trauma NT-proBNP was detected as a predictor of non-survival and especially, high level of NT-proBNP at admission or a maintaining of high levels over days indicated non-survival [33]. In the present analysis, NT-proBNP correlated positively with the previous illness Score2, the sPAP, and negatively with the left ventricle function and morphology parameters EF or LVEDD. Furthermore, in the included patients, NT-proBNP levels were significantly increased when disturbed myocardial relaxation or wall movement disorder were detected. Based on the association with the sPAP value, NT-proBNP in trauma seemed to be a marker of right ventricular dysfunction, which might be associated with high pulmonary hypertension. This is important because pulmonary hypertension must be managed in the intensive care setting. This involves right ventricular preload optimization through distinct fluid administration, diuretics, or dialysis, while the right ventricular afterload may be reduced for example by pulmonary vasodilators such as prostacyclin or phosphodiesterase type 5 inhibitors to improve right ventricular function [34]. Therefore, a systematic analysis of NT-proBNP as a marker of right ventricular function, volume load and indirect sPAP after polytrauma might be an important monitoring tool on the ICU in the future.

Interestingly both cardiac biomarkers, TnT as well as NT-proBNP were associated with the pre-existing cardiac damage risk, assessed by the Score 2. Therefore, the pre-existing conditions especially the cardiac risk profile might influence the damage extent after trauma. In the literature, there are some studies in polytrauma-patients describing the prediction of in-hospital mortality after chest trauma based on pre-existing comorbidities and their associated pharmacotherapy [35]. Further, we previously demonstrated that polytraumatized patients with cardiac comorbidities represent a separate group of patients characterized by higher concentrations of TnT, IL-33R, BMI and initial sugar levels, who therefore might need a special attention [11]. Overall, the pre-existing cardiovascular risk in trauma patients is underrepresented in the literature and requires more attention in future studies [18]. The current study highlights the relationship between trauma severity—particularly cardiac damage—and pre-existing conditions and risk factors. In non-survivors, the Score 2 was significantly higher compared to survivors, which supports the thesis that pre-existing cardiac risk factors predict the extent of post-traumatic cardiac damage. Also, the age of trauma patients, which is one factor accounting in the score 2, seems to play an important role in the development of cardiac impairment after trauma. In the supplemental Table 1, we compared the descriptive and echocardiographic data of patients < 30 years of age and > 30 years (Supplemental Table 1).

### Characterizing cardiac damage through transthoracic echocardiography: providing a comprehensive view of cardiac injury

Since laboratory markers may not fully capture cardiac dysfunction after trauma, it was important in the present study to systematically correlate these markers with a detailed analysis of cardiac function.

One of the main findings of the pilot study is the observation of the development of valvular damage/insufficiency in polytraumatized patient. Especially, the development of tricuspid insufficiency and the aggravation of the valvular dysfunction was observed (Table 2). Similar results were found earlier in a porcine polytrauma model with a liver laceration, a chest trauma, a femur fracture and a hemorrhagic shock [19]. A systematic analysis of valvular function in polytrauma patients has not been reported in the literature before, although there are some rare case reports of trauma patients developing heart valve failures. In one case report minimally invasive tricuspid valve repair was needed in a young trauma-patient after trauma before orthopaedic surgeons were able to address the other injuries [36]. Tricuspid valve injury with severe regurgitation was found to be the most common cardiac complication following blunt chest trauma [37]. The most likely reason for acute valvular regurgitation is the sudden deceleration or compression of the blood column in the heart during the vulnerable phase of the cardiac cycle [38]. In general, traumatic disruption of the chordae tendinea led to acute and severe mitral regurgitation, in case of high energetic trauma, like car accidents [39, 40]. Mitral valve dysfunction was observed in the present study in nearly 50% of patients after (day 1) and slightly increased in the second echo measurement. According to the literature, mitral valve dysfunction could manifest due pulmonary oedema or hypotension [41].

The symptoms of traumatic valvular damage can vary widely: some cases are asymptomatic for years, while others present with sudden hemodynamic instability immediately after trauma. Most symptoms typically occur within the first seven days [38, 42]. Furthermore, minimal initial valvular regurgitations can aggravate gradually over the time in hospital and can lead to the need of a surgical intervention [43]. In the present analysis of valvular regurgitations, we observed a slightly aggravation of valvular function between day 1 and day 2.

Besides a trauma induced sudden increase in intrathoracic pressure, also the treatment of patients can contribute to valvular dysfunction. In patients with decompensated heart failure and moderate to severe mitral valve regurgitation, volume overload has been shown to partially exacerbate valvular regurgitation. A reduction in volume, indicated by a decline in proBNP levels, was associated with a significant decrease in the regurgitant surface area and volume in patients with left ventricular dysfunction [44]. Therefore, volume substitution in severely polytraumatized patients may also impact their valvular function. Consequently, NT-proBNP levels might serve as an indicator of volume-related issues. Overall, valvular regurgitation was observed especially in the tricuspid valve and the mitral valve, which was not described systematically in the literature before.

We have shown that the left ventricular parameters, EF and LVEDD, minimally altered in the study patients. On the other side, diastolic function measured using E/E’ and E/A ratios indicated no significant impairment of the left ventricular diastolic function. Studies focussing on the analysis of cardiac function in polytrauma patients are very rare. At least, in a small systematic study from 1996 involving seven severely injured patients (mean ISS 38), the abnormal cardiac function post-trauma, indicated by a low fractional area change (FAC) of the left ventricle was observed [36]. In a pig polytrauma model, a significant reduction of the EF was observed within 24 h after trauma and was associated with an increase in cardiac damage markers troponin I and HFABP [3]. The literature also shows case reports of fatal damage of the left ventricle after trauma. For example, one report described a 15-year-old girl, who developed a left ventricular apical ballooning syndrome indicative of a Tako-tsubo cardiomyopathy, after a severe motor vehicle accident [45]. In patients with thermal injuries a significant reduction of the left ventricular diastolic function was observed [46].Therefore, our analysis revealed un-expectedly that left ventricular function was not altered in our polytrauma patients. Nevertheless, in the present analysis, some individual patients (belonging to non-survivors) showed a significant reduction of the EF (up to 35%) after trauma. In summary, while pronounced extend of left ventricular dysfunction is rare in polytraumatized patients, it appears to be life threatening.

Wall motion abnormalities were observed in up to 19.2% of chest trauma patients in previous literature [47], and in 25% of our polytrauma collective (Table 2). These wall movement abnormalities were accompanied with an increase of NT-proBNP, a decrease of EF and an increase of RVEDD in our patients at day two after trauma (Fig. 6A-C). In the literature, subarachnoid hemorrhage and ST segment elevation were associated with transient regional wall motion abnormalities, which shaped the term “neurogenic stunned myocardium” [48]. Cardiac damage after polytrauma is partly mediated by the mechanical component induced by chest trauma [4]. Next to increased levels of cardiac damage markers, like Troponin I/T or Heart-Fatty-Acid Binding protein (HFABP), structural damage of the heart, such as intramuscular ruptures or small bleedings, have been seen histologically in experimental polytrauma models with chest trauma [18]. Further, structural changes in cardiac tissue such as translocation of the gap junction protein Connexin 43 and the increase of cardiac tissue levels of high-mobility group box 1(HMGB-1) were found [18, 49, 50]. These structural, mechanically-induced changes were associated with impaired cardiac function in different experimental polytrauma models [18, 19, 51]. In the present study, patients with chest trauma showed an increased the IVS/LVPW ratio (Fig. 7F), and a higher E/E’ ratio (Fig. 7G) compared to patients without chest trauma. Next to direct cardiac damage, the post-traumatic inflammatory immune response has been described as a mediator of indirect cardiac damage, even in the absence of direct damage due a chest trauma [51–53]. This inflammatory response includes the release of pro-inflammatory cytokines, activation of the complement system and the release of damage associated molecular patterns (DAMPs), which have been shown in-vivo and in-vitro to impair the cardiomyocyte function by disrupting mitochondrial function, causing metabolic and structural alterations, and inducing apoptosis [54, 55]. This inflammatory-mediated damage is one reason why patients without chest trauma were also included in the present analysis. We observed, in a few cases, that massive trauma to other areas, such as extremities amputations, could lead to a significant increase in cardiac damage markers.

Additionally, we observed a disturbed myocardial relaxation in 20% of our polytrauma-patients (Table 2; Fig. 6), which was associated with an increase of NT-proBNP and TnT, as well as a decrease of the EF. Disturbed myocardial relaxation in the context of post-traumatic analysis has not been described in the literature before. Further studies in polytrauma patients are needed to bridge the literature gap.

Moreover, the present analysis highlighted the importance of right ventricular parameters. We found that TAPSE was significantly altered in our trauma patients, whereas RVEDD was slightly elevated, in accordance with a slight increase in sPAP values at days one and two after trauma. In a retrospective study with fifteen trauma patients, six patients were found to have biventricular dysfunction, one - isolated abnormal left ventricular function, and two -isolated abnormal right ventricular function in the absence of chronic obstructive pulmonary disease or pre-existing heart disease [56]. In 1988, Eddy et al. reported an association between non-survival and right ventricular dysfunction with preserved left ventricular function in multiple injured patients [57]. In the future, it is important to investigate in more details the special role of right ventricular dysfunction after polytrauma.

Altogether, based on the so far small number of included patients, not all results reached statistical significance, while showing a lot of research potential. Thereby we see a great importance in a systematic analysis of echocardiographic measurements in polytrauma patients. A key limitation of the current study is the small sample size, which will be addressed in future research by including more trauma patients and assessing their cardiac function via TTE. Additionally, these data need to be correlated with parameters such as ventilation, pulmonary hypertension, catecholamine demand and volume load in these patients. In previous studies, these parameters have been associated with a systemic increase in NT-proBNP, which was notably also elevated in the current patient cohort and increased over the course of the ICU treatment. Thereby, future NT-proBNP monitoring and echocardiographic function control, might be beneficial for polytrauma patients and lead to an improved ICU treatment.

## Conclusion

Taken together, we conducted the first systematic analysis of transthoracic echocardiography in polytraumatized patients on day one and two in polytrauma patients with an ISS ≥ 16. We observed a slightly reduced right ventricular function in these patients. Additionally, mitral and tricuspid valve regurgitations were common changes in our patient’s cohort, which might be also associated with the trauma itself, a volume overload or ventilation. In general, we observed an increase of the cardiac damage marker troponin T, as well as an increase of the heart failure marker NT-proBNP over the time. The elevated biomarkers were associated with pre-existing cardiac risk factors, the injury severity and changes in the right ventricular functions.

In the future, we aim to include additional patients in this systematic echocardiography analysis to further elucidate the role of cardiac function and especially the right ventricular dysfunction after polytrauma. Overall, the literature on cardiac function following polytrauma is outdated, with only a few well-conducted studies and case reports available. Thus, more systematic analyses of the cardiac function after polytrauma are needed to accurately delineate the full scope of cardiac dysfunction in these patients.

## Electronic supplementary material

Below is the link to the electronic supplementary material.

Supplementary Material 1

Supplementary Material 2

## Data Availability

All data generated or analysed during this study are included in this published article [and its supplementary information files].

## Abbreviations

AIS: Abbreviated Injury Scale
CRP: C-reactive protein
CVP: Central venous pressure
DGU: German Society of Trauma Surgery
DZHK: German Centre for Cardiovascular Research
EF: Ejection fraction
GCS: Glasgow Coma Scale
LVEDD: Left ventricular end diastolic diameter
LVSWI: Left ventricular stroke work index
NT-proANP: N-terminal peptide of pro-atrial natriuretic peptide
NT-proBNP: N-terminal prohormone of brain natriuretic peptide
IL-6: Interleukin-6
ISS: Injury Severity Score
IVS/LVPW: Thickness of the interventricular septum and posterior wall of the left ventricle
PT: Polytrauma
RVEDD: Right ventricular end diastolic diameter
SEM: Standard error of the mean
SF: Shortening fraction
sPAP: Systolic pulmonary arterial pressure
TAPSE: Tricuspid annular plane systolic excursion
TBI: Traumatic brain injury
TnT: Troponin T
TTE: Transthoracic echocardiography
