# Supplementary material for: Cardiac damage after polytrauma: the role of systematic transthoracic echocardiography - a pilot study
Source: World J Emerg Surg. 2025 Mar 11;20:21. doi: 10.1186/s13017-025-00596-5 (PMC11895250; doi:10.1186/s13017-025-00596-5)
Supplement: Supplementary file 1 — Supplementary Material 1 [file 13017_2025_596_MOESM1_ESM.docx]

**Supplemental Text:** Standardized Parameter measured in the systematic transthoracic echocardiography measurement (according to the German Centre for Cardiovascular Research (DZHK)).

Ultrasound quality (qualitatively)

**Left ventricle:**

- Function:
  - Systolic and diastolic (qualitatively)
    - E/E’
    - E/A
  - EF [%] (Standard value >60)
- Left ventricle:
  - Size (qualitatively)
  - Muscle thickness (qualitatively)
  - LVEDD [mm] (36-55 mm)
  - LVESD [mm] (23-40 mm)
  - IVS [mm] (≤12 mm)
  - LVPW [mm] (≤12 mm)
  - LA [mm] (20-40 mm)
- Left atrium (qualitatively)

**Right ventricle:**

- Function (qualitatively)
- Right ventricle (qualitatively)
  - TAPSE [mm]
  - RVEDD [mm] (<30 mm)
  - RVWD [mm] (<5 mm)
- Right atrium (qualitatively)

**Heart valves:**

- Aortic valve
  - Morphology (qualitatively):
    - Aortic valve opening area/plan (cm²)
    - Aortic valve opening area/cont (cm²)
    - LVOT [m m]
  - Function (qualitatively):
    - Pmax [mmHg]
    - Pmean [mmHg]
    - Aortic Insufficiency (Grade)
- Mitral valve
  - Morphology
    - Mitral valve opening area/plan (cm²)
    - Mitral valve opening area/cont (cm²)
  - Function
    - Pmax [mmHg]
    - Pmean [mmHg]
    - Mitral Insufficiency (Grade)
- Pulmonal valve (qualitatively)
- Tricuspid valve:
  - Morphology (qualitatively)
  - Function (qualitatively)
    - Mitral Insufficiency (Grade)

V. cava inferior [mm]: ZVP [mmHg]

Ascending Aorta [mm] (20-26 mm)

Pericardial effusion [mm]

Additional comments

**Summary of assessment**
