# Supplementary material for: Cardiac damage after polytrauma: the role of systematic transthoracic echocardiography - a pilot study
Source: World J Emerg Surg. 2025 Mar 11;20:21. doi: 10.1186/s13017-025-00596-5 (PMC11895250; doi:10.1186/s13017-025-00596-5)
Supplement: Supplementary file 2 — Supplementary Material 2 [file 13017_2025_596_MOESM2_ESM.docx]

Supplementary Table S1. Comparison of cardiac parameters between younger (<30 years) and older (>30 years) patients.

| Parameter | <30 years (Mean value ± SD) (n=7) | >30 years (Mean value ± SD) (n=28) | P value |
| --- | --- | --- | --- |
| Age | 23.9 ± 2.85 | 61.9 ± 14.9 | <0.0001**** |
| ISS | 26 ± 10 | 30 ± 9.1 | n.s. |
| Score 2 | 2.28 ±1.12 | 10.4 ± 11.04 | 0.005 |
| Non-Survivors | 28.6% | 28.6% |  |
| Contusio cordis | 0 | 21.4% |  |
| Chest trauma | 42.9% | 71.4% |  |
| Previous Coronary Artery Disease | 0 | 14.3% |  |
| Previous Arrhythmia | 0 | 10.7% |  |
| Arterial Hypertension | 0 | 50% |  |
| Past myocardial infarction | 0 | 14.3 % |  |
| Troponin T (ER) | 105 ± 211 | 33 ± 43 | n.s. |
| Troponin T (d1) | 252 ± 518 | 129 ± 156 | n.s. |
| Troponin T (d2) | 101 ± 180 | 72 ± 73 | n.s. |
| NT-proBNP (ER) | 50.7 ±86.2 | 708 ± 1575 | 0.03* |
| NT-proBNP (d1) | 233 ± 324 | 894 ± 1750 | n.s. |
| NT-proBNP (d2) | 784 ± 813 | 810 ± 1070 | n.s. |
| EF [%] (d1) | 65 ± 0 | 60.5 ± 9.4 | n.s. |
| EF [%] (d2) | 66 ± 3.75 | 60.8 ± 10.8 | n.s. |
| LVEDD [mm] (d1) | 62 ± 0 | 47 ± 7.2 | 0.03* |
| LVEDD [mm] (d2) | 49 ± 8.1 | 47 ± 6.1 | n.s. |
| IVS/LVPW [mm] (d1) | 0.9 ± 0 | 1.3 ± 0.3 | n.s. |
| IVS/LVPW [mm] (d2) | 1 ± 0.2 | 1.2 ± 0.2 | n.s. |
| TAPSE [mm] (d1) | 24± 0 | 24 ± 3.9 | n.s. |
| TAPSE [mm] (d2) | 28 ± 3.4 | 28 ± 7.9 | n.s. |
| RVEDD [mm] (d1) | 22± 0 | 25 ± 7.6 | n.s. |
| RVEDD [mm] (d2) | 21 ± 2.3 | 29 ± 3.7 | 0.004** |
| E/E’ (d1) | 4± 0 | 7.8 ± 2.4 | 0.02* |
| E/E’ (d2) | 5.5 ± 2.8 | 7.5 ± 2.4 | n.s. |
| E/A (d1) | 1.2 ± 0 | 1.2 ± 0.4 | n.s. |
| E/A (d2) | 1.3 ± 0.3 | 1.5 ± 0.5 | n.s. |
| sPAP [mmHg] (d1) | n.a. | 32 ± 14 | n.a. |
| sPAP [mmHg] (d2) | 8 | 29 ± 11 | n.s. |

n.s. = not significant, n.a. = not available; d1 = day 1, d2 = day 2


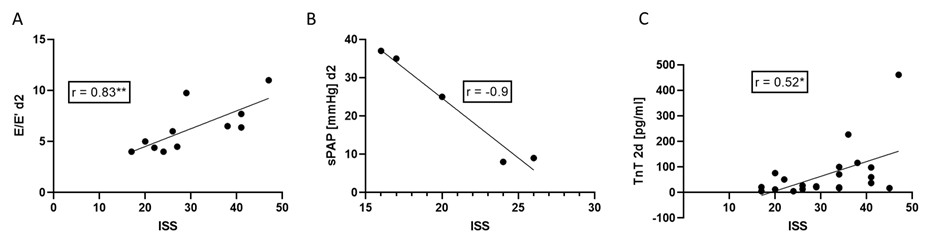


Supplemental Figure 1:

Supplemental Figure 1: **Further correlation analysis with the Injury Severity Score (ISS).** A) Strong Correlation (r= 0.83; CI -0.5747 to 0.9230) between the E/A ratio at day 2 after Trauma. B) Correlation analysis between the ISS and the systolic pulmonary arterial pressure (sPAP) in mmHg at day 2. (CI -0.9988 to -0.7412) C) Moderate Correlation (r = 0.52; CI 0.1054 to 0.7845) between the injury severity and the troponin T concentration (TnT) at day 2 after polytrauma with an ISS of ≥16. *p≤0.05, Spearman rank correlation.
